# The Resting Brain Sets Support-Giving in Motion: Dorsomedial Prefrontal Cortex Activity During Momentary Rest Primes Supportive Responding

**DOI:** 10.1093/texcom/tgaa081

**Published:** 2020-11-02

**Authors:** Tristen K Inagaki, Sasha Brietzke, Meghan L Meyer

**Affiliations:** Department of Psychology, San Diego State University, San Diego, CA, USA; Department of Psychological and Brain Sciences, Dartmouth College, Hanover, NH 03755, USA; Department of Psychological and Brain Sciences, Dartmouth College, Hanover, NH 03755, USA

**Keywords:** default network, prosocial behavior, resting state, social cognition, social support

## Abstract

Humans give support, care, and assistance to others on a daily basis. However, the brain mechanisms that set such supportive behavior in motion are unknown. Based on previous findings demonstrating that activity in a portion of the brain’s default network—the dorsomedial prefrontal cortex (DMPFC)—during brief rest primes social thinking and behavior, momentary fluctuations in this brain region at rest may prime supportive responding. To test this hypothesis, 26 participants underwent functional magnetic resonance imaging (fMRI) while they alternated between deciding whether to give support to a close other in financial need, receive support for themselves, and make arbitrary decisions unrelated to support. Decisions were interleaved with brief periods of rest. Results showed that, within participants, spontaneous activity in the DMPFC during momentary periods of rest primed supportive-responding: greater activity in this region at the onset of a brief period of rest predicted, on a trial-by-trial basis, faster decisions to give support to the close other. Thus, activating the DMPFC as soon as our minds are free from external demands to attention may help individuals “default” to support-giving. Implications for understanding the prosocial functions of the resting brain are discussed.

## Introduction

Support-giving behaviors, ranging from providing emotional and physical care to offering financial assistance, are pervasive social behaviors. Support-giving appears throughout the life span, emerging in the first year of life and continuing through adulthood (Weiner and Graham 1989; Zahn-Waxler et al. 1992; Warneken and Tomasello 2006, 2007). In the United States of America, people report supporting others every day, whether it be caring for a parent or child, listening to a spouse’s frustrations with a colleague, or helping a friend through a tough time (American Time Use Survey et al. 2019). Such behavior serves critical functions for humans, ensuring infant survival (Bowlby 1988) and potentially leading to better emotional, social, and physical health for the support giver (Brown and Brown 2006; Inagaki 2018). Therefore, processes that facilitate such a critical social behavior may be built into human brain function. In other words, the ubiquity and benefits of support-giving raises the question of whether there may be brain mechanisms in place that set supportive responding in motion.

To date, the majority of neuroscience research on support-giving focuses on neural responses to caring for others in need. This literature implicates brain regions associated with primary rewards (i.e., the ventral striatum [VS]) as well as the regions implicated in parental care-giving (i.e., septal area [SA]) in supporting others (Inagaki 2018). For example, in nonhuman animals, providing support to offspring is associated with increased activity in the VS (Stack et al. 2002), whereas lesions to either the VS or the SA severely disrupt supportive behavior (Slotnick and Nigrosh 1975; Hansen 1994). In humans, these regions have also been implicated in giving support to romantic partners, friends, and family members in multiple contexts, including physical pain (Inagaki and Eisenberger 2012) as well as financial need (Inagaki et al. 2016; Inagaki and Ross 2018). Yet, while these findings help identify which brain mechanisms “respond” to support-giving, they do not answer the question of whether there may be brain mechanisms that set this supportive behavior in motion. This gap is surprising, given past suggestions that supportive behavior often occurs instinctively with little deliberation (Preston 2013; Zaki and Mitchell 2013). What neural mechanisms facilitate this default tendency to give support?

An answer to this question may stem from the observation that a brain region consistently associated with other-focused cognition—the dorsomedial prefrontal cortex (DMPFC)—is also reliably engaged during brief rest (Shulman et al. 1997; Raichle et al. 2001; Schilbach et al. 2008). An established body of neuroimaging research demonstrates that when left unprompted by external stimuli or directions, a specific network of brain regions spontaneously increases activity (Shulman et al. 1997; Raichle et al. 2001; Buckner et al. 2008). While this “default network” encompasses multiple brain regions (Andrews-Hanna, Reidler, Sepulcre, Poulin, & Buckner, 2010; Yeo et al. 2011), the DMPFC region of this network is of particular interest, given its role in other-focused social cognition. That is, in addition to activating by default during rest, the DMPFC shows reliable increases in activity when participants are instructed to consider other people’s thoughts, emotions, and traits (Saxe and Kanwisher 2003; Mitchell et al. 2004; Harris et al. 2005; Frith and Frith 2006; Van Overwalle 2009; Spunt et al. 2011).

Relevant to the inclination to give support, recent findings suggest that DMPFC activity, as soon as our minds are free from external demands to attention, may facilitate other-focused behavior. Specifically, greater activity in the DMPFC at the onset of a brief rest trial predicts faster responding on subsequent trials that require considering another person’s point-of-view (Spunt et al. 2015; Meyer and Lieberman 2018). For example, in one brain imaging study (Spunt et al. 2015), participants alternated between randomly presented trials in which they had to make decisions (by pressing a button) that either did or did not require judging another person’s mental state. A briefly presented rest trial (~6 s) also occurred prior to each decision. Trial-by-trial analyses revealed that greater activity in the DMPFC at the onset of the prior rest period predicted faster responding to subsequent mental state inference trials. These findings are consistent with other neuroscience research findings that spontaneous, prestimulus neural activity facilitates stimulus responding (Linkenkaer-Hansen et al. 2004; Fox et al. 2006; Hsieh et al. 2012; Brooks et al. 2013) and fit with the theoretical basis of priming from cognitive and social psychology (Neely 1977; Higgins 1989). The premise of priming is that activating a given representation, such as of an “apple,” makes one faster to next detect (i.e., primes) related representations, such as “banana” (Neely 1977; Higgins 1989; Tulving and Schacter 1990). To the extent that default activity in DMPFC at rest involves mental operations relevant to other-focused thought, then by analogy, DMPFC activity at rest may be an endogenous prime, making one faster to consider another person’s perspective. Indeed, this interpretation aligns with previous suggestions that automatic and spontaneous neural processes may increase the efficiency of subsequent, related responding (Lindquist 2013; Barrett 2017).

Given that support-giving is a ubiquitous and important form of other-focused behavior, default activity in the DMPFC may likewise prime supportive responding. Although no research to date has tested this hypothesis, two pieces of evidence hint to this possibility. First, research on altruism, in which participants make other-focused decisions at a cost to the self (Moll et al. 2006; Harbaugh et al. 2007), has found that greater DMPFC activity while participants are instructed to consider people’s mental states in the scanner correlates with the amount of money donated to (unrelated) charities outside of the scanner (Waytz et al. 2012). Second, greater functional connectivity between the DMPFC and other default network regions during extended rest was found to positively relate to individual differences in support-giving, both at the time of the scan and during a follow-up measure collected 1 month later (Inagaki and Meyer 2019). Moreover, this association appeared to be relatively specific to support-giving. DMPFC connectivity at rest was associated with giving support but not with receiving support, and the association between DMPFC connectivity and support-giving remained statistically significant after adjusting for extraversion, a more general measure of engaging in social interaction. Collectively, these two sets of findings implicate the DMPFC, both during other-focused cognition and extended rest, in support-giving. Yet, whether spontaneous DMPFC activity at rest primes support-giving remains untested.

Here, we assessed whether DMPFC activity during momentary rest primes subsequent support-giving. If this is the case, then greater spontaneous activity in this region prior to opportunities to give support should predict faster decisions to do so. To test this possibility, participants underwent functional magnetic resonance imaging (fMRI) while they played a raffle game in which they believed they were collecting raffle tickets for themselves and, in a second condition, forgoing tickets for themselves in order to give tickets to a close other in financial need, with both the participants and their close others who earned tickets entered into a raffle for a cash prize. Decisions were interleaved with brief rest periods (2–6 s). To the extent that activating the DMPFC by default during rest primes supportive-responding, greater activity in this region at the onset of a given rest period should predict—trial-by-trial—a faster decision to give raffle tickets to the close other.

## Materials and Methods

### Participants

Thirty-two participants (*M* age = 19.2, standard deviation [SD] = 0.98, 23 females; 15 White/Caucasians, 11 Asians, 4 African Americans, and 2 Hispanics) screened for contraindications for the MRI environment (nonremovable metal in the body, claustrophobia, and pregnancy) were run in the current study. Participants had the choice to receive cash payment or course credit in exchange for their participation and were entered into a raffle for an additional cash payment at the end of the study (see Support-Giving Scanner Task). Participants provided written informed consent in accordance with the Dartmouth College Institutional Review Board.

Sample size was determined a priori following a power analysis in fMRI power (fmripower.org; Mumford and Nichols 2008) using the comparison of giving > neutral decisions from previously published studies (Inagaki et al. 2016; Inagaki and Ross 2018) and the same VS Region-of-Interest (ROI) as used in the present study. Results of this analysis suggested that 25–35 participants would yield at least 80% power to detect a small effect size (Cohen’s *d* between 0.30 and 0.35) in the VS at a *P* < 0.05. Due, in part, to financial constraints, data collection ended once 32 participants had been collected to guard against data loss due to motion or potential outliers in the behavioral data (see below). The current task was run concurrently with a task aiming to test a separate theoretical question (Brietzke and Meyer, unpublished data).

### Support-Giving Scanner Task

We optimized a commonly used support-giving task (Moll et al. 2006; Inagaki et al. 2016; Inagaki and Ross 2018), so that it would allow us to test our hypothesis that spontaneous activity at rest primes support-giving behavior. Prior to beginning the task, participants were asked to “select someone [they] know who is in financial and emotional need. For example, this person could have mounting student loan debt and may also be going through relationship or health issues.” Participants chose to play for friends (73.077%), family members (23.077%), and romantic partners (3.846%), all rated as close others (*M* = 88.38, SD = 12.004, range = 61–100).

In the scanner, participants played a raffle game in which they had the opportunity to win raffle tickets for the close other (giving condition) and for themselves (receiving condition). We chose to assess giving to a close other because of the types of support-giving behavior that humans engage in, giving to close others (friends, family, spouses, and children), especially when those individuals are in need, is arguably the most common and meaningful (Preston 2013; Inagaki and Orehek 2017; Inagaki 2018; Inagaki and Ross 2018).

After the completion of the entire study, raffle tickets were placed into a drawing for a $200 cash prize. Thus, the more raffle tickets the participant collected for their close other and, separately, for themselves, the greater each of those individuals’ chances were of winning the prize. To heighten the believability that the decisions participants made would have real consequences for the close other, participants were asked to think of someone who needed money and were also instructed they would later have to provide the contact information for their close other in the event that they won the raffle. A large canister of raffle tickets also appeared in the scanner console room to further enhance believability.

Following previous iterations of the same task (Inagaki et al. 2016; Inagaki and Ross 2018), the support-giving task consisted of three experimental trial types: offers to give tickets while forgoing tickets collected for the self (i.e., giving condition), receive tickets for themselves without any cost to the close other (i.e., receiving condition), and offers in which neither the participant nor the close other collected tickets (i.e., an arbitrary decision; neutral condition). As in prior work (Inagaki et al. 2016; Inagaki and Ross 2018), the range of tickets possibly won for the self or other in a given trial ranged between 10 and 70. The relative cost to the self of giving to the close other was evenly distributed between smaller trade-offs (i.e., 10–20 tickets) and larger trade-offs (i.e., 30–40 tickets) in order to keep participants engaged in the task.

**Figure 1:**
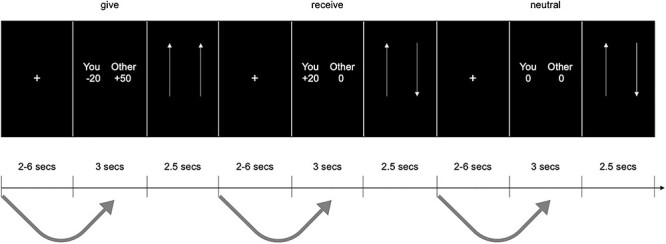
Experimental paradigm. Decisions to give support to a close other, receive for the self, and make arbitrary decisions unrelated to support (i.e., neutral decisions) were interleaved with brief periods of jittered rest. In addition, attention-orienting trials, in which participants indicated if two arrows pointed in the same direction, followed decisions in order to reduce continued thinking about the previous decision. Analyses assessed whether the activity at the onset of the rest period preceding an offer predicted the reaction time to accepting that offer.

Participants had up to 3 s to make their decision to accept or reject the offer by pressing a 1 (accept) or 2 (reject) button on a scanner-safe button box. As soon as participants made their decision, the screen advanced to an attention-orienting trial in which participants had up to 2.5 s to indicate whether the two arrows on the screen were oriented in the same or different directions (Fig. 1) after which the screen advanced to a rest period of jittered fixation (range = 2–6 s, *M* = 2.96, SD = 0.86). The purpose of the attention-orienting trials was to help ensure that participants cleared their minds before the subsequent rest period and is based on prior default network priming research (Meyer and Lieberman, 2018). Participants responded to 160 offers (80 giving, 40 receiving, and 40 neutral) over two runs of scanning and the trial types were shown in a randomly presented order. We oversampled giving trials based on the pilot data from past work showing that participants accept the receiving trials at a higher rate than giving trials; since we are interested in giving trials specifically, oversampling helped ensure a more equitable distribution of accepted giving and accepted receiving trials. Three participants did not complete their scan session (*n* = 1 due to claustrophobic feelings, *n* = 2 due to scanner hardware malfunction), three individuals were excluded from analyses (accepted too few giving offers for reliable statistical modeling, i.e., accepted fewer than 16 trials), and another participants’ parameter estimates from the DMPFC ROI was more than 3 SDs outside of the group’s mean, leaving a final sample of 26. Note that this sample size is still above the predetermined cutoff of 25 participants.

### Postscan Measures

After exiting the scanner, the participants completed questionnaires assessing (1) their own and their close other’s level of emotional and financial need and (2) the supportive feelings they experienced during the support-giving task. Ratings were made using a 0–100 scale anchored by “Not at all” and “Most possible.” Emotional and financial need were assessed with the items: How much emotional/financial need is this person (are you) in these days? Supportive feelings in response to the task were assessed with the items previously related to giving support to a close other, including feelings of social connection, support effectiveness, and desire to help (Inagaki and Eisenberger 2012). Specifically, participants were instructed to think back to the times when they chose to get tickets for the person they know and report on how that decision made them feel: How connected did you feel to this person? How effective do you think this decision could be for this person? and How much did you want to help this person?

### fMRI Data Acquisition

Imaging took place on a Siemens Prisma 3T scanner. Functional images in response to the support-giving task were acquired using an EPI gradient-echo sequence (2.5 $\times$ 2.5 $\times$ 2.5 mm voxels, TR = 1000 ms, TE = 30 ms, 2.5 mm slice thickness, FOV = 24 cm, matrix = 96 $\times$ 96, flip angle = 59${}^{\circ}$; simultaneous multislice [SMS] = 4). A T2-weighted structural image was acquired coplanar with the functional images (0.9 $\times$ 0.9 $\times$ 0.9 mm voxels, TR = 2300 ms, TE = 2.32 ms, 0.9 mm slice thickness, FOV = 24 cm, matrix = 256 $\times$ 256, flip angle = 8${}^{\circ}$).

### Data Analyses

#### Brain Imaging Data Preprocessing

We preprocessed our brain imaging data with fMRIprep version 1.4.0 (Esteban et al. 2018). For each subjects’ two functional runs of the support-giving task, the following preprocessing was performed. First, a reference volume and its skull-stripped version were generated using a custom methodology of fMRIPrep. The BOLD reference was then coregistered to the T1w reference using register (FreeSurfer), which implements boundary-based registration (Greve and Fischl 2009). Coregistration was configured with nine degrees of freedom to account for distortions remaining in the BOLD reference. Head-motion parameters with respect to the BOLD reference were estimated using MCFLIRT (FSL 5.0.9, Jenkinson et al. 2002). This step generated six motion regressors (corresponding to rotation and translation parameters) that were used in our first-level models to control for participant motion. BOLD runs were slice-time corrected using 3dTshift from AFNI (Cox and Hyde 1997). The BOLD time-series were resampled to MNI152NLin2009cAsym standard space, generating a preprocessed BOLD run in MNI152NLin2009cAsym space. After preprocessing, first- and second-level statistical analyses were performed in SPM12 (Wellcome Department of Cognitive Neuroscinece, Institute for Neurology, United Kingdom).

#### Support-Giving Task Activation Models for Each Subject

Before testing our primary hypothesis that prestimulus activity in the DMPFC primes support-giving, we ran a general linear model for each subject to assess neural activity in response to each task condition. Consistent with prior work using this task (Inagaki and Ross 2018), we assessed neural responses to accepted giving trials, accepted receiving trials, and neutral trials. The rationale to only assess accepted giving and receiving trials is (1) that they are the only trial types in these conditions with adequate statistical power and measurement reliability, given that on average 92.5% (SD = 20.6%) of receiving trials and 75.185% (SD = 19.4%) of giving trials were accepted (see statistical comparisons of decision types in Results) and (2) that they are the only trials in which support-giving behavior can be assessed. Task trials were modeled as a boxcar function, from the onset of the offer until the participant’s button response to the next attention-orienting trials, and the rest periods served as the implicit baseline in these models. That said, it is noteworthy that the results are identical if the attention-orienting trials are included in the modeling of the implicit baseline and only the giving, receiving, and neutral decision are modeled as task-related activity. Regressors in our model included: accepted giving trials, accepted receiving trials, neutral trials, rejected giving/receiving trials, and six motion regressors of no interest.

#### Neural Priming Models for Each Subject

Following past work assessing DMPFC priming (Spunt et al. 2015; Meyer and Lieberman, 2018), our priming analyses followed two additional steps at the subject level. These steps we applied to the task-activation general linear model in which responses to each task condition was modeled as a boxcar beginning at the onset of a trial and extending until the end of the follow-up attention-orienting trial and included separate regressors for the accepted giving, accepted receiving, neutral, rejected giving/receiving trials, and six motion regressors. First, for each subject, the residual images from this general linear model were saved and all subsequent priming analyses were performed on these residual images. We are therefore able to assess neural activity during prestimulus rest that is statistically independent of the neural activity associated with the task itself.

Second, we ran a parametric modulation analysis on the residual images. In this parametric modulation analysis, there were three conditions: accepted giving trials, accepted receiving trials, and neutral decision trials. For these conditions, we modeled the onset of the prestimulus rest period preceding each of these offer trial types. Each of these conditions included a parametric modulator representing the reaction time, in seconds, with which the participants made their response on the offer trial following the preceding rest period. For example, for the giving trial shown in Figure 1, the onset of the rest trial before the option to give was modulated by the speed with which the participant subsequently decided to accept that giving offer. Also consistent with past DMPFC priming research (Spunt et al. 2015; Meyer and Lieberman, 2018), the onset of the rest period was modeled as a “punctate response,” with a duration of 0. This approach further ensures that prestimulus activity unrelated to the extensive thinking about future trials is assessed. Evidence of priming is negative activation, as greater activation at the onset of a rest trial should predict faster (i.e., numerically smaller) reaction times. The use of reaction time as our primary outcome follows the use of reaction time in the priming literature (Higgins 1989; Neely 1977; Tulving and Schacter 1990). Finally, we also ran follow-up analyses to ensure neural activity associated with priming support-giving, which could not be explained simply by the duration of rest (2–6 s) preceding a given trial. To this end, we also ran an analysis in which the duration of the rest period was included as the first parametric modulator, followed by trial reaction time as the next parametric modulator. Because parametric modulators were orthogonalized, any observed priming effect in these models controls for the variance explained by the rest duration itself.

#### ROI Definition

**Figure 2:**
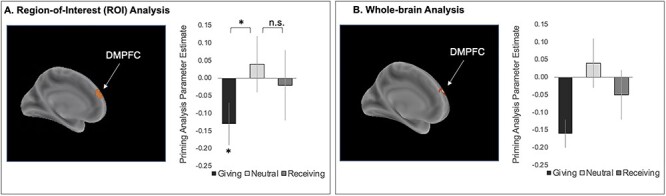
DMPFC activity primes decisions to give. (*A*) The DMPFC region-of-interest based on the cluster identified by Spunt et al. (2015) and the priming parameter estimates from this ROI for each experimental condition. (*B*) The DMPFC cluster (identified by a whole-brain search) that primes faster decisions to give support to the close others and the priming parameter estimates from this cluster for each experimental condition. Statistical testing on these parameter estimates were only conducted for the ROI analysis to ensure statistical independence between identified and tested voxels. Thus, parameter estimates in (*B*) are for visualization only. Error bars reflect within-subjects standard errors. ^*^ = *P* < 0.05, two-tailed and a BCa 95% CI excluding 0. Negative parameter estimates indicate greater priming.

Following our previous theoretical model (Inagaki 2018), VS and SA ROIs that have been shown to increase in response to similar versions of the current task were assessed in response to the present study’s giving, receiving, and neutral conditions. Bilateral VS ROIs were structurally defined by combining the caudate and putamen from the Automated Anatomical Labeling atlas (Tzourio-Mazoyer et al. 2002) and by constraining the regions at −24 < *x* < −24, 4 < *y* < 18, and − 12 < *z* < 0 (Inagaki and Eisenberger 2012; Inagaki et al. 2016; Inagaki and Ross 2018), and a previously defined ROI of the SA (Zahn et al. 2009) that also relates to support-giving was combined into a single masked ROI reflecting the brain voxels associated with giving support. To test our DMPFC priming hypothesis, we created a 10-mm sphere ROI based on the peak reported by Spunt et al. (2015), who identified a DMPFC cluster specifically associated with both prestimulus activity during brief rest and social cognition (DMPFC: *x* = −9, *y* = 57, and *z* = 30; Fig. 2*A*). Significance was determined based on a *P* value of 0.05, two-tailed or a bias corrected and accelerated (BCa) bootstrap 95% confidence interval (CI) excluding 0. Data can be found on the Open Science Framework: https://osf.io/3k6va/.

#### Whole-Brain Analyses

We followed-up our ROI analyses with whole-brain analyses to further assess neural activity that primes giving. Whole brain, group analyses were conducted on each participant’s first-level models. Parametric modulation analyses measuring within-condition priming were assessed at the second level as a *t*-contrast and condition comparisons for the parametric modulation analyses were compared in a flexible factorial design. We assessed our whole-brain results with a threshold of *P* < 0.005, family-wise error (FWE) corrected cluster size for each contrast, which identified the following cluster extent thresholds across contrasts: *k* > 149 voxels for within-condition priming analyses, and *k* > 156 voxels for flexible factorial comparisons between priming conditions.

## Results

### Behavioral Results

We first examined whether the participants’ perceived need of their close other and themselves were equitable. There were no significant differences between how much financial or emotional need the participants thought their close other was in (financial *M* = 48.920, SD = 24.113; emotional *M* = 59.460, SD = 27.051) relative to themselves (financial *M* = 45.85, SD = 29.463, *t*(25) = 0.531, *P* = 0.600, BCa 95% CI = [−7.373, 13.206]; emotional *M* = 58.650, SD = 28.050, *t*(25) = 0.125, *P* = 0.902, BCa 95% CI = [−11.926, 12.423]). Thus, any potential differences in the reaction time between giving and receiving conditions cannot be attributed to differential need between the self and the close other.

As a manipulation check that the task elicited the intended support-giving experience, we next examined the associations between the perceived need and feelings previously shown to increase after giving support to a close other, specifically feelings of social connection and support effectiveness (Inagaki and Eisenberger 2012; Inagaki and Ross 2018). As expected, the greater perceived financial and emotional need of the close other was associated with higher feelings of social connection with the close other when participants chose to give support to them (financial need: *r* = 0.391, *P* = 0.048, BCa 95% CI = [0.020, 0.623]; emotional need: *r* = 0.549, *P* = 0.004, BCa 95% CI = [0.152, 0.775]). Perceived financial need of the close other was trending toward an association with greater support effectiveness (*r* = 0.323, *P* = 0.108, BCa 95% CI = [−0.036, 0.567]). Taking into account the type of support-giving manipulated in the current study (i.e., financial assistance), the perceived emotional need of the close other was, unsurprisingly, not significantly related to support effectiveness (*r* = 0.273, *P* = 0.177, BCa 95% CI = [−0.195, 0.613]).

Next, decisions to give and receive were evaluated. Participants accepted a higher percentage of receiving trials (*M* = 92.212%, SD = 2.090%) than giving trials (*M* = 74.664%, SD = 1.957%; *t*(25) = 3.376, *P* = 0.002, BCa 95% CI = [0.260, 0.087]). Further, consistent with past work using a similar task (Moll et al. 2006), participants were faster to accept raffle tickets for themselves (*M* = 0.926 s, SD = 0.208) than they were to give raffle tickets to the close other (*M* = 1.184 s, SD = 0.309, *t*(25) = 5.986, *P* < 0.001, BCa 95% CI = [0.159, 0.352]). However, within the opportunities to give support to a close other, participants were faster to accept than to reject offers to give (*M* = 1.422 s, SD = 0.335, *t*(25) = 3.807, *P* < 0.001, BCa 95% CI = [0.374, 0.119]).

### Neural Results

#### VS and SA Activity in Response to Support-Giving

Before assessing our primary hypotheses about DMPFC priming support-giving, we examined the VS and SA activity in response to giving. Although there were no differences between conditions with the ROI approach (*t*s < 0.770, *Ps* > 0.300, BCa 95% CI range = [−0.566, 0.572]), activity in the VS and SA were related to the individual differences in the desire to help the close other. Specifically, greater desire to help was associated with greater average VS and SA activity to giving to a close other (vs. neutral, *r* = 0.442, *P* = 0.024, BCa 95% CI = [0.035, 0.698]). There was no association, however, between the desire to help the close other and the VS and SA activity to receiving (vs. neutral, *r* = 0.125, *P* = 0.542, BCa 95% CI = [−0.352, 0.533]). Additionally, and consistent with the idea put forth by past work that both offer types may be relatively “rewarding” (Moll et al. 2006; Harbaugh et al. 2007; Inagaki and Eisenberger 2012), the whole-brain analysis showed that giving and receiving (vs. neutral) decisions were associated with a dorsal striatum cluster that extended ventrally, with a peak in VS (*x* = 18, *y* = 6, *z* = −2); see Supplementary Table 1.

#### DMPFC Activity Primes Support-Giving

The primary aim of the present study was to assess whether DMPFC activity at rest primes subsequent support-giving decisions. We first assessed this possibility with a DMPFC ROI defined based on another study, which specifically identified a DMPFC cluster associated with increased activity in response to both (1) brief rest and (2) social cognition (Spunt et al. 2015). Consistent with our prediction, this DMPFC ROI primed support-giving (*t*(25) = 2.281, *P* = 0.031, BCa 95% CI = [−0.252, −0.037]; Fig. 2*A*), and this result remained significant even when the length of a given rest period was controlled for (i.e., added as a parametric modulator; *t*(25) = 2.531, *P* = 0.018, BCa 95% CI = [−0.258, −0.044]. That is, greater DMPFC activity at the onset of a rest period predicted faster reaction time on the subsequent decision to give. Further, directly comparing the parametric modulation analyses for the DMPFC between the conditions again showed stronger priming of giving (vs. neutral) decisions (*t*(25) = 2.239, *P* = 0.034, BCa 95% CI = [−0.317, −0.009]; Fig. 2*A*) but no significant difference in priming the receiving (vs. neutral) decisions (*t*(25) = 0.470, *P* = 0.642, BCa 95% CI = [−0.258, 0.185]).

Given the results reported above, we next considered the possibility that DMPFC priming has implications for brain activity in response to the decisions to give to a close other. Therefore, we ran an exploratory correlation analysis between the DMPFC ROI priming effect of giving and VS and SA ROI activity in response to giving (vs. neutral) decisions. Interestingly, individuals who showed the strongest DMPFC priming giving effect also showed the greatest neural activity in the VS and SA in response to giving (*r* = −0.324, *P* = 0.107, BCa 95% CI = [−0.632, −0.028]). The negative correlation indicates that the more the DMPFC of the dorsomedial subsystem primes the decision to give support (i.e., a numerically smaller parameter estimate), the more activation in response to giving support.

#### Whole-Brain Analyses

Searching across the whole brain for evidence of priming revealed findings consistent with our ROI results. The whole-brain parametric modulation analysis assessing which regions’ activity at the onset of a rest period predicted faster decisions to give revealed a single cluster in the DMPFC (Table 1, Fig. 2*B*), and this cluster remained significant even when the length of a given rest period was controlled for (i.e., added as a parametric modulator). Moreover, directly comparing the parametric modulation analysis for giving (vs. neutral) decisions showed that the DMPFC cluster identified in the giving parametric modulation analysis remained significant, albeit at a slightly smaller voxel extent (*k* = 138), and no other clusters emerged in this comparison. Indeed, the parametric modulation analysis of the receiving and neutral decisions indicated that no regions of the brain showed evidence of priming either of these decisions. Similarly, no portions of the brain showed preferential priming for the receiving (vs. neutral) decisions. Collectively, our results suggest that the DMPFC may play a particularly strong role in priming supportive decisions.

**Table 1:** Whole-brain analysis assessing brain regions that prime decisions to give

	Region	*x*	*y*	*z*	*t*	*k*
Regions associated with priming decisions to give	DMPFC	−18	50	26	4.40	221
		−6	56	32	3.11	

Note: The only region to prime decisions to give was a single cluster in the DMPFC. No clusters in the brain were identified as priming either receiving or neutral decisions. Activations were significant at *P* < 0.005, 149 voxels. Coordinates are in the Montreal Neurological Institute (MNI) space; *t* = *t* statistic value at peak coordinates; and *k* = cluster voxel extent.

## Discussion

Of all human social behavior, giving care and support to others is among the most common. Even without external cues, reminders, or reward, we often instinctively give to others even when there are costs to the self (Preston 2013; Zaki and Mitchell 2013). The current study aimed to determine whether this may be the case, in part, because spontaneous activation in the DMPFC during brief periods of rest primes support-giving behavior a mere few seconds later. Consistent with this hypothesis, DMPFC activity during brief rest was associated with faster subsequent decisions to give support to a close other in need. Specifically, within the set of participants’ decisions to give, prestimulus DMPFC activity—measured with ROI and whole-brain levels of analysis—predicted faster subsequent giving decisions. Collectively, our findings provide a parsimonious mechanism to help explain why support-giving is such a ubiquitous (American Time Use Survey et al. 2019) and instinctual (Zaki and Mitchell 2013) behavior.

Given the importance of support-giving behavior for individual survival early in life, the maintenance of close social relationships, and long-term health and well-being (Bowlby 1988; Feeney and Collins 2001; Inagaki 2018), it may not be a coincidence that natural fluctuations in neural activity, even during very brief rest, would prime support-giving. Indeed, it has been proposed that default network activity at rest may help coordinate processes relevant for survival (Buckner et al. 2008), including social connection (Mitchell 2006; Schilbach et al. 2008; Lieberman 2013; Meyer 2019). Previously, it has been shown that the DMPFC primes social inferences about others—for example, helping individuals interpret other people’s mental states and personality traits (Spunt et al. 2015; Meyer and Lieberman 2018). The current results complement and extend previous findings to suggest that prestimulus activity in the DMPFC, a key node of the default network, primes supportive decisions to help a close other in need and establishes a potential mechanism by which humans evidence such frequent, spontaneous prosocial behavior. Interestingly, the DMPFC showed no evidence of priming the nonsupport-giving decisions and in the whole-brain search, no regions outside of the DMPFC primed support-giving. This further speaks to the potentially specific role of the DMPFC in priming supportive behavior.

Our findings add important, novel insight into the possible mechanism that links the tendency to engage the default network during extended rest to prosocial behavior. To date, most studies relating the default network at rest to positive social outcomes correlate (1) resting state functional connectivity (i.e., time-course correlations of neural activity) between default network regions over the course of several minutes with (2) variables related to positive social interactions (Kennedy and Courchesne 2008; Weng et al. 2010; Dodell-Feder et al. 2014). For example, resting state functional connectivity between the DMPFC and other default network regions during extended rest correlates with trait empathy (Dodell-Feder et al. 2014) and prospectively predicts greater self-reported support-giving (Inagaki and Meyer 2019). Yet, this literature persists without a clear explanation as to how engaging these regions during rest directly impacts prosocial behavior. This is due, in large part, to the fact that the default network is assessed in these studies for several minutes and social behavior is measured outside of the MRI scanner. We overcame this barrier by intermixing brief rest with prosocial decisions in a single fMRI paradigm to assess how momentary fluctuations in the DMPFC at rest impact prosocial responding, finding that greater DMPFC activity at the onset of rest (i.e., as soon as participants had a mental break from the experimental task) predicts faster support-giving directly after. To our knowledge, these results provide the first explanation as to why default network engagement during rest corresponds with prosocial outcomes.

While our results are consistent with the hypothesis that the DMPFC primes support-giving that is intended to be helpful, it is also possible that this region primes additional other-focused behaviors that are less prosocial. For example, it has been suggested that prioritizing the gains of ingroup members, such as close others, may correspond with the desire to derogate outgroup members (Iyengar et al. 1999; Waytz and Epley 2012; Meyer et al. 2015). Might the DMPFC therefore also prime decisions to give unsupportive responses to outgroup members? Additional research that includes opportunities to punish (e.g., give harm, as opposed to support) or make other social decisions that vary along the dimensions of both prosociality and the recipients’ ingroup/outgroup status are needed in order to assess the specificity of our results to support-giving. In addition, conditions in which participants have the opportunity to give support to social targets in varying levels of need, or more abstract causes, such as giving to charities instead of a close other, will further clarify the conditions under which the brain at rest primes supportive behavior.

Another interesting direction for future research will be to assess whether prestimulus DMPFC activity not only makes one faster to give support but also predicts whether or not a supportive decision is made. It should be noted that the current task is designed to encourage a high percentage of giving by asking participants to select a close other who they identified as in need of financial support. And indeed, participants in our study decided to make supportive decisions (i.e., increase the chances of their close other winning money) on 75% of giving trials, a choice distribution consistent with past work with this task (Inagaki et al. 2016; Inagaki and Ross 2018). Therefore, we did not have enough variability in participant choices to assess if the DMPFC primes whether or not a supportive decision is made. Given that priming research specifically focuses on decision speed (Neely 1977; Higgins 1989; Tulving and Schacter 1990), our findings fit within the priming framework. That said, another study assessed altruistic giving by asking participants to forgo winning money for themselves in order to win money for a stranger and observed more variability in giving decisions (Hutcherson et al. 2015). This allowed the researchers to assess reaction time and the decision to give (or not) simultaneously. Interestingly, they found that DMPFC activity over the course of decisions, in addition to other default network regions (i.e., ventral medial prefrontal cortex [VMPFC] and tempoparietal junction [TPJ]), contributed to the combination of the choice to give (vs. not) and the speed with which participants made this choice. Future default network priming research that assesses giving towards strangers or close others in varying levels of need may help determine whether the prestimulus activity in DMPFC also primes the combination of the choice to give and the speed with which this decision is made.

It is noteworthy that during the task itself, we found that VS and SA showed equitable increases in activity in response to giving and receiving, whereas previous research finds that VS and SA activity in response to giving is greater than receiving (Moll et al. 2006; Telzer et al. 2014; Inagaki et al. 2016; Inagaki and Ross 2018). One possible explanation could be that the perceived needs of the close other and the participant were equivalent in the current study. Nonetheless, we still observed that the self-reported desire to help the close other positively and differentially correlated with VS and SA activity in response to giving (vs. receiving) decisions, which is consistent with past work implicating these regions in promoting care for others. Indeed, we also observed that, on average across giving decisions, the DMPFC priming of the giving decisions positively correlated with activity in VS and SA in response to the giving decisions. This points to the possibility that the priming effects generated by the resting brain may link to the reinforcing nature of support-giving. Though we encourage caution in our interpretation as (1) the *P* value suggested a trending association despite the fact that the CI indicated a significant correlation (i.e., the CI did not include 0) and (2) the motivation for examining this correlation was exploratory. An interesting direction for future research, should the association replicate with a larger sample, may be to characterize the temporal relationships—on a moment-by-moment basis—between the dorsomedial subsystem priming giving and the subsequent reward-value of giving to better understand how these two timepoints may reinforce one another.

Beyond implications for prosocial behavior, there is a growing appreciation for the contribution of support-giving to the link between social support and physical health (Inagaki 2018). Giving more support to others is associated with better health outcomes (e.g., Piferi and Lawler 2006; Moieni et al. 2019) even when adjusting for receiving support (Brown et al. 2003). Further, giving to a close other in need (vs. a control condition where no support is given) reduces stress-related physiological responding to an acute stressor, suggesting that giving to others may also influence health via reductions in stress (Inagaki and Eisenberger 2016). DMPFC engagement during rest has also been associated with measures of inflammation, a key biological mechanism linking social behavior with disease (Marsland et al. 2017). Greater resting-state connectivity between the DMPFC and other default network regions are associated with less inflammation (i.e., circulating plasma levels of interleukin 6 [IL-6]). However, previous research on task-induced DMPFC engagement actually finds that this region is associated with negative health-related outcomes (Eisenberger et al. 2007; Dedovic et al. 2009). For instance, stronger functional connectivity between the DMPFC and amygdala in response to negative social evaluation is related to a higher, rather than lower, inflammatory response to the evaluation (IL-6, Muscatell et al. 2015). Differences in when the DMPFC is measured (rest vs. task) as well as the type of social cognition paradigm employed (support-giving vs. social evaluation) make it difficult to speculate on the precise role of DMPFC support-priming and subsequent health effects. However, future research integrating measures of the brain at rest prior to opportunities to give support, as studied here, with inflammatory or other health-relevant outcomes may clarify the role of the DMPFC in support-giving’s effects on health.

In conclusion, we found the first evidence for the novel possibility that the DMPFC primes support-giving. Within a given participant, greater default DMPFC activity at rest predicted faster subsequent decisions to help a close other in need. The current findings add to a growing literature on the social functions of spontaneous DMPFC activity. More broadly, given that the DMPFC is among the regions of the default network that activate when we stop attending to external stimuli, these findings suggest that disengaging from the external environment—even briefly—may facilitate our instinctive prosocial nature.

## Supplementary Material

SupplementaryMaterialfinal_tgaa081

## Notes

We would likea to thank Frances Sperry for assisting with data collection. *Conflict of Interest*: None declared.
