# Supplementary material for: The Resting Brain Sets Support-Giving in Motion: Dorsomedial Prefrontal Cortex Activity During Momentary Rest Primes Supportive Responding
Source: Cereb Cortex Commun. 2020 Nov 2;1(1):tgaa081. doi: 10.1093/texcom/tgaa081 (PMC8152835; doi:10.1093/texcom/tgaa081)
Supplement: SupplementaryMaterialfinal_tgaa081 [file supplementarymaterialfinal_tgaa081.docx]

The resting brain sets support-giving in motion:

dorsomedial prefrontal cortex (DMPFC) activity during momentary rest primes supportive responding

Tristen K. Inagaki, Sasha Brietzke, & Meghan L. Meyer

Supplementary Material

Table 1

*Clusters activated in response to Support-Giving Task*


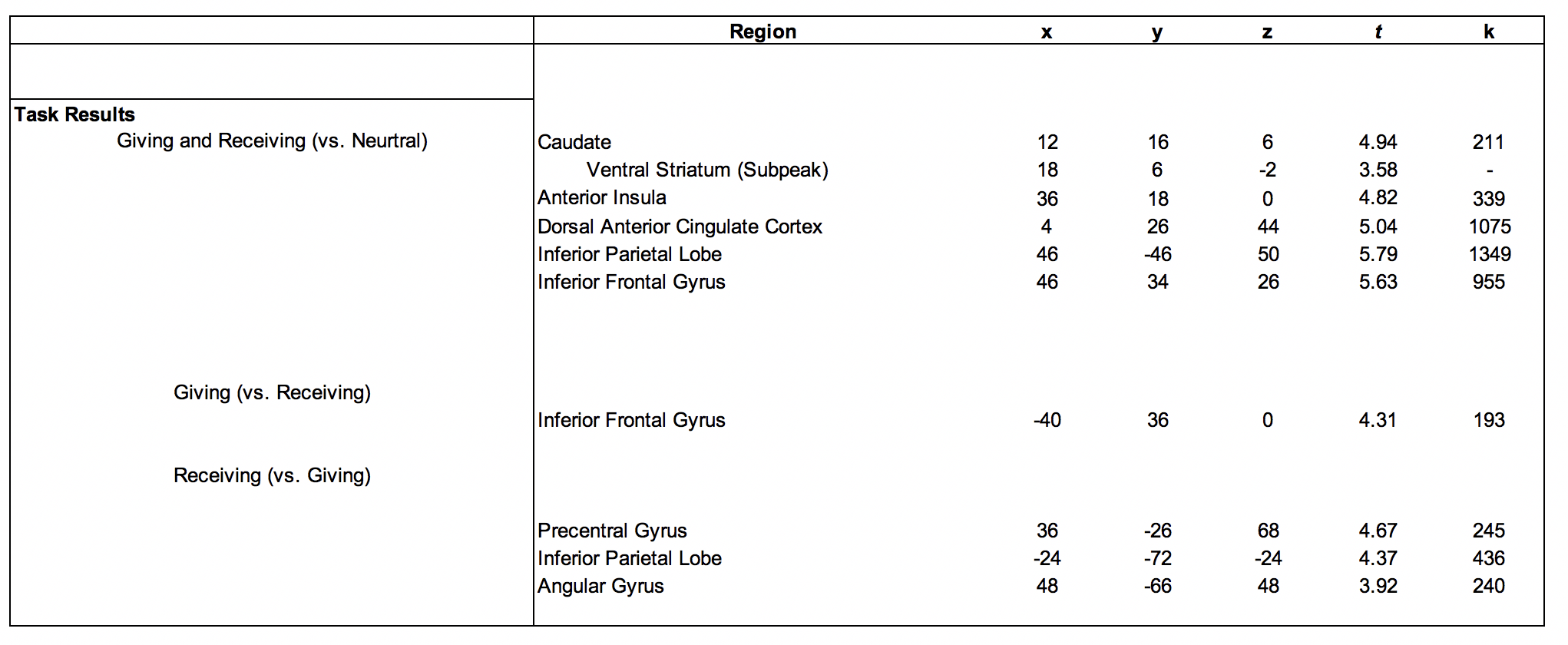


*Note.* Activations significant at *p* < .005, family-wise-error (FEW) corrected cluster size of 192 voxels. Coordinates in Montreal Neurological Institute (MNI) space; *t* = t statistic value at peak coordinates; *k* = cluster voxel extent
